# Surgical Outcomes of Olecranon Osteotomy Approach Combined With Submerged Kirschner Wires and Plate Fixation for Duckerley IIIB Distal Humeral Coronal Shear Fractures

**DOI:** 10.1111/os.70005

**Published:** 2025-02-19

**Authors:** Zhou‐Feng Song, Wei‐Qiang Zhao, Zeng‐Li Zhang, Jie‐Feng Huang

**Affiliations:** ^1^ Department of Orthopaedics & Traumatology The First Affiliated Hospital of Zhejiang Chinese Medical University Hangzhou Zhejiang China; ^2^ The First Clinical College, Zhejiang Chinese Medical University Hangzhou Zhejiang China; ^3^ Department of Orthopaedics & Traumatology Songyang Hospital of Traditional Chinese Medicine Songyang Zhejiang China; ^4^ Department of Orthopaedics & Traumatology Qingyuan Hospital of Traditional Chinese Medicine (Qingyuan Branch Hospital of Zhejiang Provincial Hospital of Chinese Medicine) Qingyuan Zhejiang China

**Keywords:** capitellum and trochlea fractures, coronal shear fractures, distal humerus, management, surgical approach

## Abstract

**Objective:**

Duckerley type IIIB distal humerus fractures are rare and complex injuries that pose significant challenges in both diagnosis and treatment. Currently, no consensus exists on the fixation method, with existing approaches often struggling to handle small fragments and associated with issues like elbow instability. The purpose of this study is to evaluate the surgical outcomes of submerged Kirschner wires combined with plate or submerged screw fixation technique for the treatment of Duckerley type IIIB distal humerus fractures.

**Methods:**

A retrospective analysis was conducted on 10 patients with Duckerley type IIIB distal humerus fractures who were treated at our hospital from February 2017 to April 2021. The treatment involved applying buried Kirschner wires combined with microplate or buried screw fixation technique through the olecranon osteotomy approach. The study included six males and four females, with a mean age of 51.4 ± 15.34 years (ranging from 22 to 69 years). During the follow‐up, the elbow range of motion, Mayo Elbow Performance Score (MEPS), American Shoulder and Elbow Surgeons (ASES) score, and complications were assessed.

**Results:**

All 10 patients received regular clinical and imaging follow‐up for a mean of 39.7 ± 8.8 months (range: 25–50 months). Postoperative incision healing was good for all patients, and no neurovascular injuries were noted. Two patients developed elbow pain. At the last follow‐up before the internal fixation removal operation (9.6 ± 1.9 months), X‐ray and CT findings confirmed bony healing, and no internal fixation loosening and breakage occurred in any of the patients, except for one case in which there was displacement of the Kirschner wires. The mean range of motion of the elbow before the internal fixation removal operation was extension 15.0° ± 21.6°, flexion 129.5° ± 28.1°, pronation 83.0° ± 9.2°, and supination 81.5° ± 8.0°. The MEPS score was 83.0 ± 8.3, and the ASES was 83.6 ± 7.8. At the last follow‐up, the mean range of motion of the elbow was extension 10.0° ± 21.9°, flexion 133.5° ± 16.0°, pronation 88.0° ± 11.2°, and supination 85.0° ± 9.5°. The MEPS score was 84.6 ± 7.6, and the ASES was 84.1 ± 7.4.

**Conclusions:**

The treatment of Duckerley type IIIB low distal humerus fractures using submerged Kirschner wires combined with plate or submerged screw fixation technique has satisfactory advantages in terms of fracture reduction, maintenance of the position of internal fixation, and postoperative recovery.

## Graphical abstract

1. This study addresses the management of a rare and challenging fracture type (Duckerley type IIIB distal humeral coronal shear fractures), providing valuable insights and a surgical approach that may improve clinical outcomes.
2. The results indicate that this technique provides satisfactory fracture reduction, stable internal fixation, and favorable functional recovery.

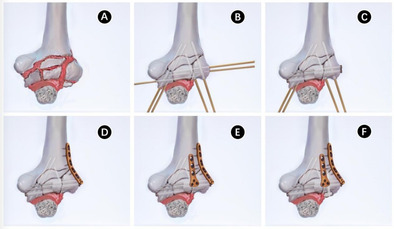

## 1 Introduction

A low distal humerus fracture, also known as a coronal shear fracture (CSF), is a rare and complex fracture involving the humeral tuberosity and/or the trochanter, occurring with a probability of approximately 1% of all elbow fractures and 3%–6% of distal humerus fractures [1, 2]. The mechanism behind low distal humerus fractures remains unclear. It is generally believed that these fractures are caused by direct axial impact of the radial tuberosity on the distal humerus when the elbow joint is in a state of semi‐flexion or hyperextension at the time of the injury. This type of fracture is often accompanied by lateral collateral ligament injury and radial head fracture, making fracture management challenging [3].

**FIGURE 1:**
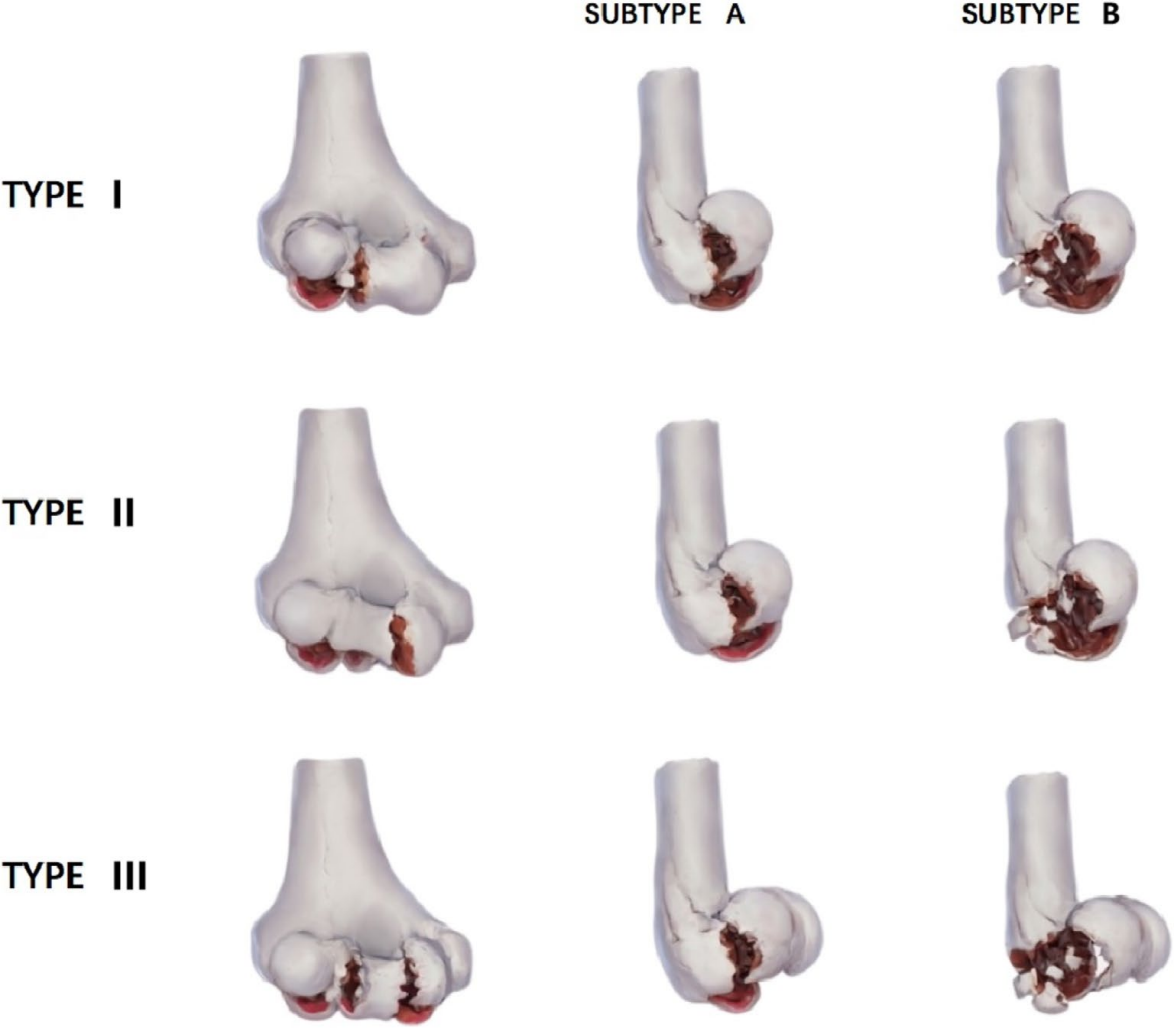
Dubbrey's classification.

Duckerley et al. [4] proposed a new classification system based on fracture anatomic pattern, number of fragments, and degree of fracture healing to guide surgical decision making and to benefit the surgeon. The fractures were classified into three types: Type 1 included humeral tuberosity fractures with or without involvement of the lateral ramus of the trochanter; Type 2 included fractures of the humeral tuberosity and trochanter as a single unit; and Type 3 included fracture separation of both the humeral tuberosity and trochanter. Each type was further divided into two subtypes based on the presence (Subtype B) or absence (Subtype A) of posterior fracture comminution (Figure 1). Duckerley type IIIB fractures pose a significant surgical challenge due to the severe and relatively rare comminution of the fracture block. Currently, there is no universally accepted method of fixation [5, 6, 7].

The existing fixed methods include nonslip plates, headless compression screws, Kirschner wires, and sutures [6, 8, 9]. The Herbert screw is widely used to fix fracture fragments due to its superior fixation strength and its ability to be embedded beneath the articular surface, allowing for early mobilization. However, fixing smaller fracture fragments remains challenging. Regarding the surgical approaches, to achieve better visualization of the anterior area, an additional lateral window is often required. This is achieved by detaching the lateral epicondyle of the humerus, the anterior capsule, and the brachialis muscle from their respective attachments [10]. Some scholars have advocated for triceps‐preserving approaches, such as the paraspinal approach or the paraspinal triceps approach, which are considered suitable for distant arthroplasties [5, 10]. However, there is still a lack of large‐sample follow‐up studies to determine whether these approaches are superior to the falciform osteotomy approach overall [11, 12].

This study describes a technique of submerged Kirschner wires combined with microplates or submerged screw fixation for patients with Duckerley type IIIB distal humerus fractures. The purposes of this study are as follows: (i) to evaluate the effectiveness of this surgical treatment; (ii) to analyze the clinical, functional, and radiological outcomes following the procedure; and (iii) to investigate the occurrence of complications and assess the long‐term efficacy and safety of this surgical technique.

## 2 Materials and Methods

Ethics committee approval was received for this study from the Institutional Review Board of the First Affiliated Hospital of Zhejiang Chinese Medical University (No. 2024‐KLS‐689‐02). We conducted a retrospective study on patients who underwent diagnosis and surgical treatment of Duckerley type IIIB distal humerus fractures at our hospital from February 2017 to April 2021.

The study included patients who met the following criteria: (i) a clear diagnosis of Duckerley type IIIB distal humerus fractures based on clinical history and imaging examination; (ii) closed fracture within 2 weeks of injury; (iii) normal function of the affected limb before injury; (iv) indications for surgery and informed consent of patients and their families to the treatment plan; and (v) willingness to follow the doctor's recommendations for postoperative rehabilitation training and willingness to accept follow‐up visits.

Exclusion criteria for this study include: (i) open fractures combined with injury to other parts of the ipsilateral upper limb or with osteofascial compartment syndrome; (ii) old or pathologic fractures; (iii) the presence of mental or psychological disorders or any other conditions that may prevent or hinder cooperation with the follow‐up visit; and (iv) if the affected limb has a history of previous surgery, infections, rheumatoid arthritis, traumatic arthritis, elbow deformity, or other forms of arthritis, such as psoriasis, Charcot's joint, and other lesions, it is important to consider these factors.

All patients underwent x‐rays, computed tomography (CT) and three‐dimensional reconstruction to determine compliance with Duckerley type IIIB and were operated on by the same group of surgeons. Clinical and imaging follow‐up was conducted for all patients (Table 1).

**TABLE 1:** Epidemiologic data and follow‐up results of patients.

Patient	Gender	Age (years)	Mechanism of injury	Extension/flexion/pronation/supination (°)		MEPS score	ASES score	Complications	Follow‐up (months)
				Pre‐removal	Last follow‐up	Pre‐removal	Last follow‐up		
1	Male	22	Accident	10/135/85/85	10/140/85/85	77/80	77/79	Elbow pain	50
2	Female	54	Fall from the ground	5/130/90/85	5/130/90/85	95/95	96/98	None	48
3	Female	69	Fall from the ground	5/120/90/85	5/125/95/90	80/80	83/83	Elbow pain	47
4	Male	54	Fall from height	5/140/85/90	5/140/85/90	83/85	82/83	None	45
5	Male	35	Accident	0/140/90/90	0/145/90/90	95/97	93/97	None	45
6	Male	60	Fall from height	5/125/90/90	5/130/95/90	83/85	82/85	None	39
7	Male	49	Sport	10/135/80/90	10/135/85/85	85/85	86/86	None	38
8	Female	67	Fall from the ground	70/90/60/65	70/95/65/70	70/70	73/73	Elbow pain	32
9	Female	65	Assault	0/135/90/75	0/140/95/75	87/89	88/92	None	28
10	Male	39	Accident	5/135/90/75	0/135/85/80	85/85	86/86	None	25

### 2.1 Surgical Method

**FIGURE 2:**
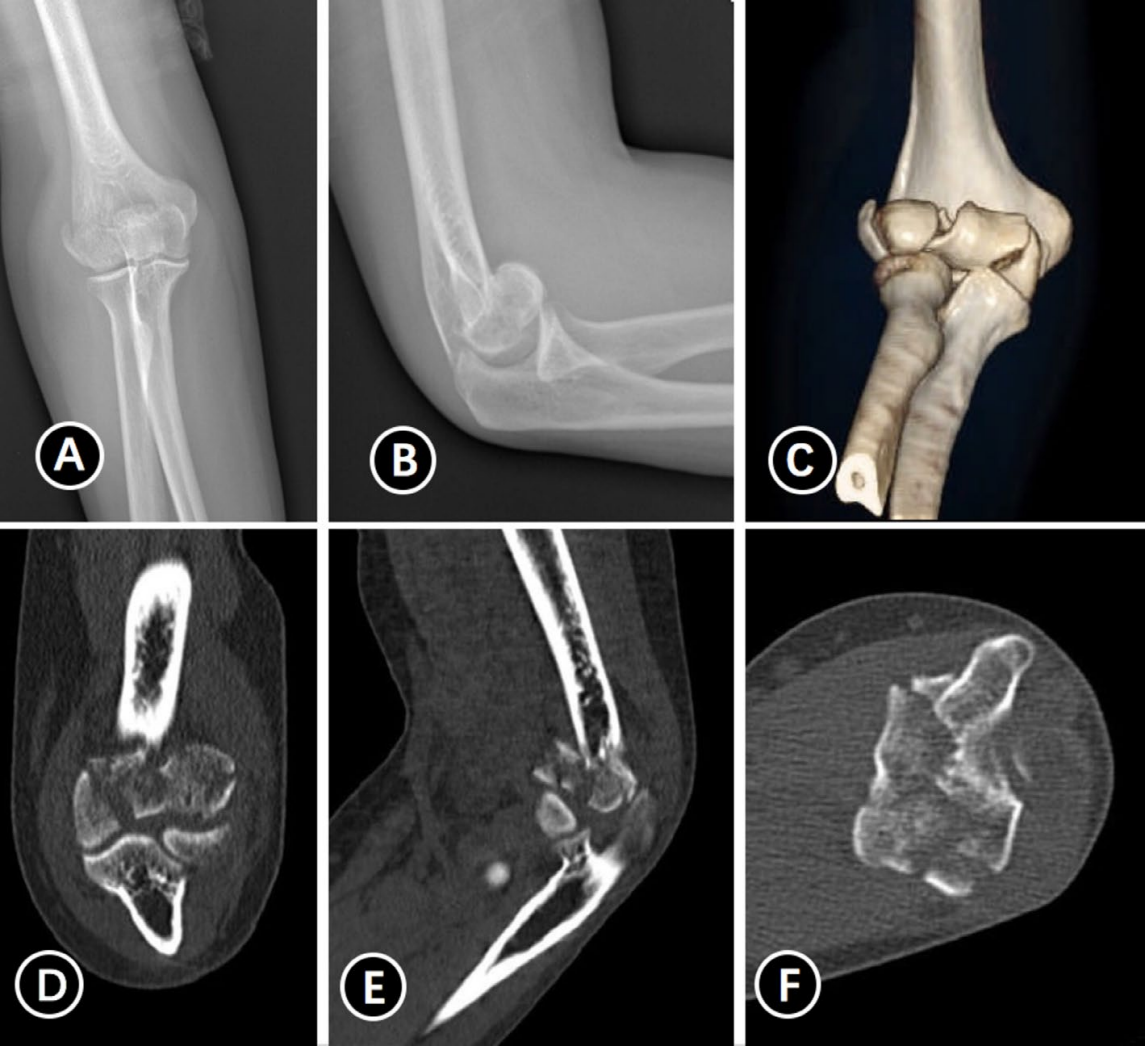
Preoperative radiography and CT examination and three‐dimensional construction of a 54‐year‐old female with Duckerley type IIIB distal humerus fractures (A–F).

**FIGURE 3:**
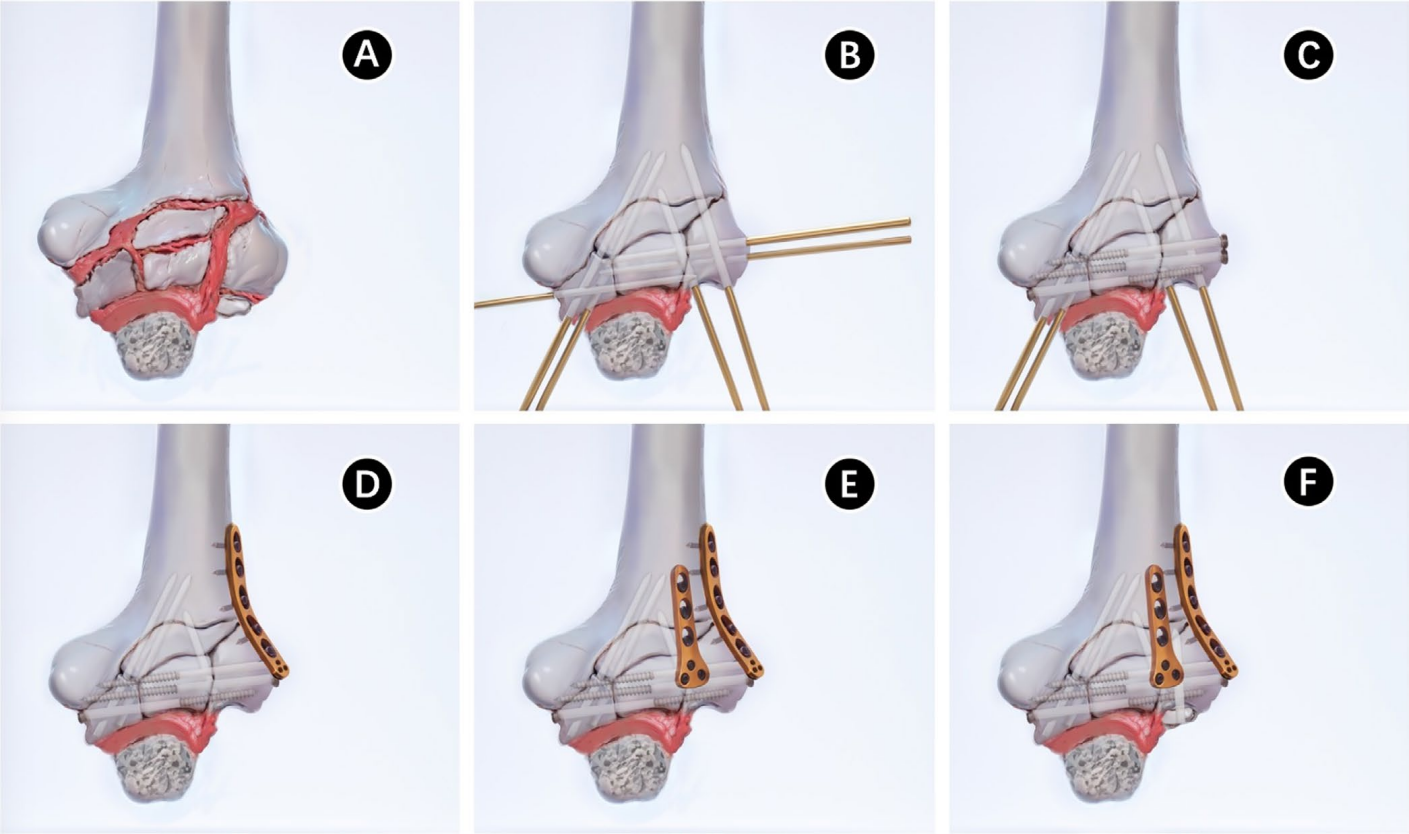
Operation diagram. (A) Postolecranon osteotomy showed distal humerus fracture. (B) After resetting the larger fracture fragments and selecting the appropriate Kirschner wires for temporary fixation based on the size of the fracture block. (C) Countersunk screws were used instead of Kirschner wires for fixation. (D, E) One to two surgical plates were placed on the dorsal aspect of the joint to resist the shear forces at the fracture site. (F) The tail of Kirschner's needle is placed below the surface of the cartilage to secure smaller fracture fragments that cannot be fixed with countersunk screws.

**FIGURE 4:**
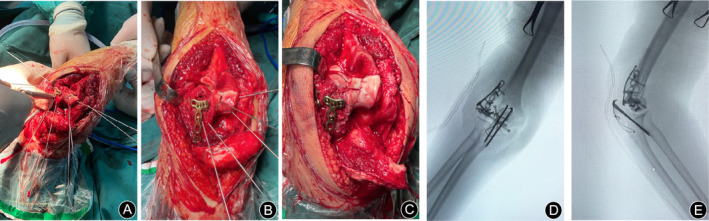
Actual picture of surgery. (A–C) The fracture was fixed with submerged Kirschner wires combined with microplates and submerged screw fixation during the surgery. (D, E) The pictures of the C‐arm machine during the surgery.

All cases were operated on under general anesthesia in the supine position. Taking Figure 2 as an example, to improve visualization of the articular surface of the distal humerus, we used the olecranon osteotomy approach to complete all procedures, followed by thorough irrigation to remove any blood clots. After resetting the larger fracture fragments and selecting the appropriate Kirschner wires for temporary fixation based on the size of the fracture block, one to two surgical plates were placed on the dorsal aspect of the joint to resist the shear forces at the fracture site. Subsequently, countersunk screws were used instead of Kirschner wires for fixation. For smaller fragments that could not be fixed with countersunk screws, the technique of placing the tail of the Kirschner needle below the surface of the cartilage was used instead of removing the fragment [13] (Figures 3 and 4). The range of motion and stability of the elbow joint are assessed by passive mobilization to ensure that the implant does not impede joint motion. Additionally, anchors are used to repair any ligaments that require attention. Anterior surgical maneuvers on the ulnar nerve are not routinely performed unless there is entrapment of the nerve after repositioning and fixation [14].

### 2.2 Postoperative Management

To prevent ossifying myositis, the patient was administered indomethacin orally postoperatively [15, 16]. A brace was used to immobilize the patient's elbow in a 90° position for 6 weeks.

Patients were followed up at 4 weeks, 6 weeks, 3 months, 6 months, 9 months, 12 months, 24 months, and then yearly for clinical, functional, and radiological evaluations. After undergoing the surgical intervention, the elbow remained immobilized in a brace for 6 weeks. Following this, patients began active and passive range of motion exercises. After fracture healing, internal fixation removal (except countersunk screws and Kirschner wires) was performed (time of surgery: 9.6 ± 1.9 months). We did not routinely release the elbow when removing the internal fixation.

### 2.3 Imaging and Clinical Functional Assessment

Radiological examinations, including x‐rays and CT and three‐dimensional reconstruction, were conducted on the first postoperative day. Two attendings completed the imaging evaluations. The Mayo Elbow Performance Score (MEPS) [17] and the American Shoulder and Elbow Surgeons (ASES) score [18] were used for clinical functional assessment, recording elbow flexion, extension, and forearm rotation. Radiographic assessment was also performed to evaluate fracture repositioning, fracture healing, osteoarthritis, ossifying myositis, and other occurrences.

### 2.4 Statistical Analysis

Counting data were expressed with the mean ± SD (standard deviation); a significant Statistical analysis was processed using SPSS software (version 26.0; Chicago, IL, USA). The range of motion, complications, follow‐up duration, and functional assessment outcomes were analyzed.

## 3 Results

### 3.1 Follow‐Up and Clinical Characteristics

All 10 patients received regular clinical and imaging follow‐up, with a mean follow‐up of 39.7 ± 8.8 (25–50) months. There were 6 (60%) more males than 4 (40%) females, and the mean age was 51.4 ± 15.34 (22–69) years.

### 3.2 Postoperative Recovery and Complications

**FIGURE 5:**
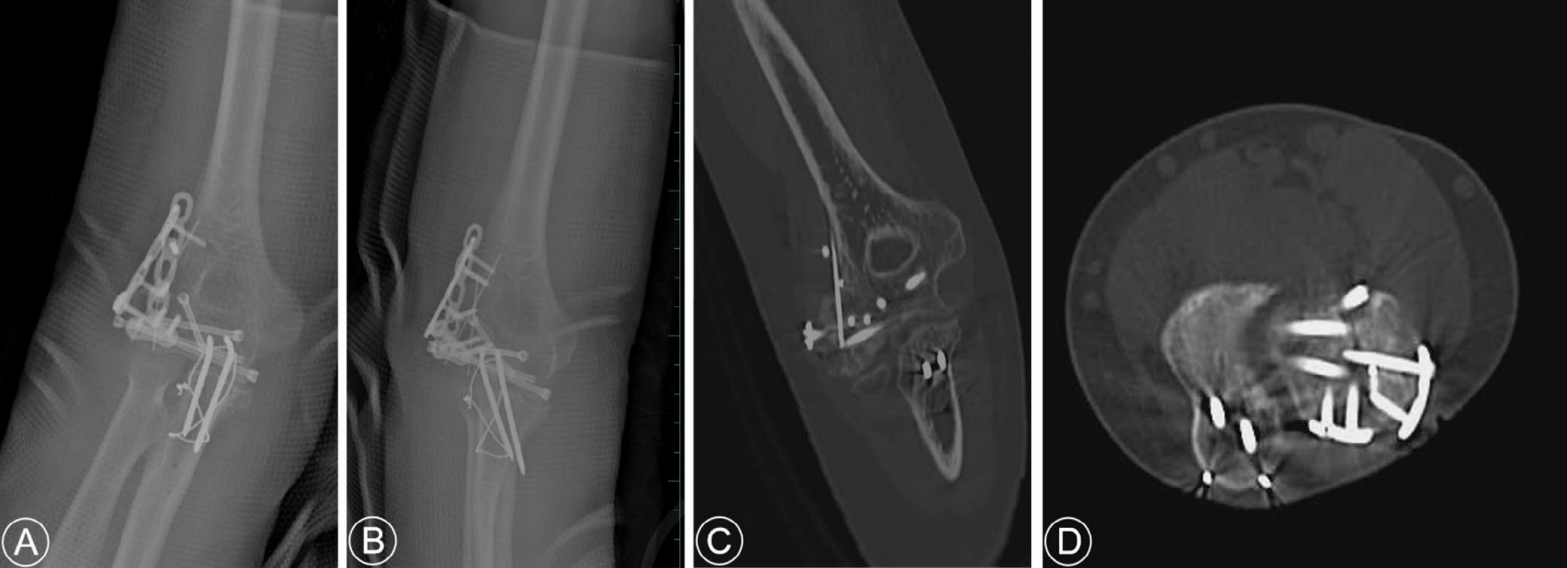
Postoperative image. (A, B) Two pictures of postoperative x‐rays. (C, D) Coronal and sagittal images in CT scans.

**FIGURE 6:**
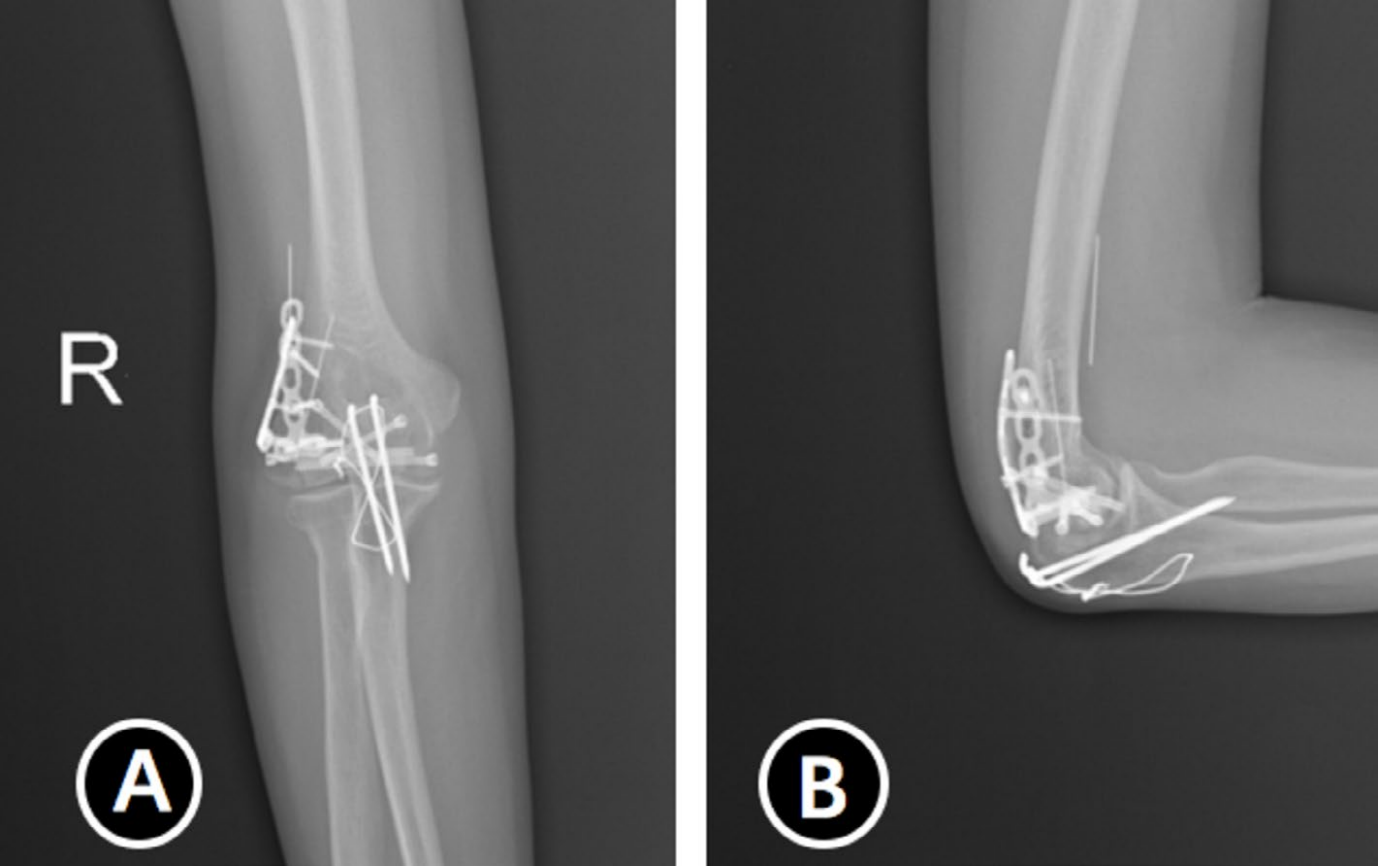
(A, B) Displacement of Kirschner wire at 9 months postoperatively, and the fracture has healed.

**FIGURE 7:**
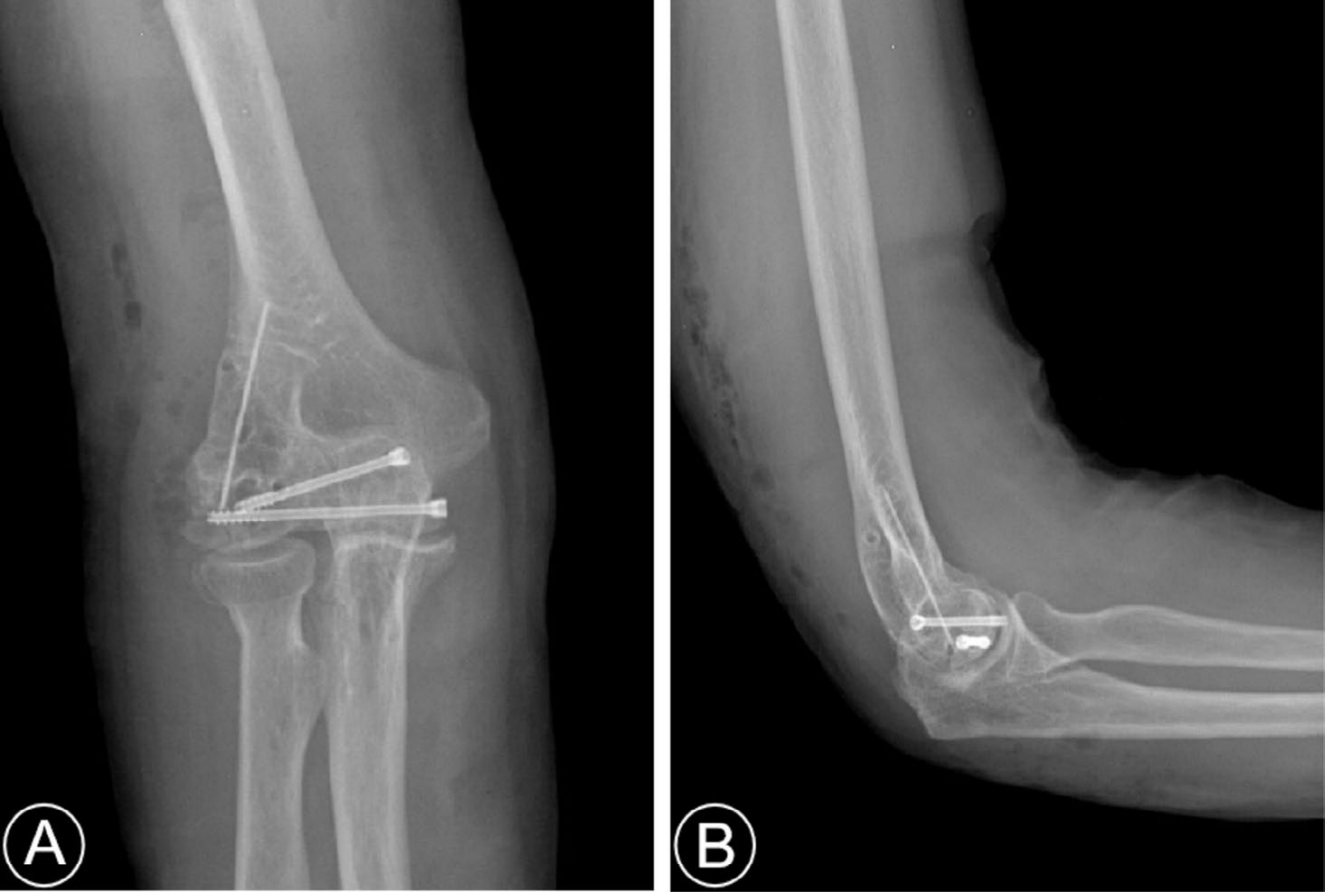
(A, B) A partial removal of internal fixation (including dislocated Kirschner wire) at 11 months postoperatively.

All patients had good postoperative incision healing, and no neurovascular injuries were noted. Two patients developed elbow pain. Before the internal fixation removal operation, x‐ray and CT findings confirmed bony healing, and no internal fixation loosening and breakage occurred in any of the patients (Figure 5), except for one case that experienced displacement of the Kirschner wires (Figures 6 and 7).

### 3.3 Elbow Range of Motion and Functional Scores

The mean range of motion (ROM) of the elbow before internal fixation removal was as follows: extension 15.0° ± 21.6°, flexion 129.5° ± 28.1°, pronation 83.0° ± 9.2°, and supination 81.5° ± 8.0°. The mean MEPS was 83.0 ± 8.3, and the mean ASES score was 83.6 ± 7.8.

At the final follow‐up, the mean ROM of the elbow improved to: extension 10.0° ± 21.9°, flexion 133.5° ± 16.0°, pronation 88.0° ± 11.2°, and supination 85.0° ± 9.5°. The mean MEPS score increased to 84.6 ± 7.6, and the mean ASES score improved to 84.1 ± 7.4.

## 4 Discussion

### 4.1 Summary of Main Findings

In this study, we evaluated the surgical outcomes of submerged Kirschner wires combined with plate or submerged screw fixation technique for the treatment of Duckerley type IIIB distal humerus fractures. The results showed that this approach provided satisfactory fracture reduction, good internal fixation, and favorable postoperative recovery. The mean range of motion at the last follow‐up improved significantly, with a MEPS score of 84.6 ± 7.6 and an ASES score of 84.1 ± 7.4. Most patients showed considerable improvement in both range of motion and functional scores, highlighting the effectiveness of the surgical treatment in restoring elbow function following complex fractures.

Distal humerus fractures are more common in individuals over the age of 60, particularly in women due to a greater angle of carry and osteoporosis [19, 20]. In our study, the mean age of patients was 51.4 ± 15.34 years, with a higher incidence in males who experienced high‐energy injuries, while females experienced low‐energy injuries. This finding contradicts other studies on the epidemiology of distal humerus fractures. It is possible that this difference is related to the scope of the present study, which only included patients with Duckerley type IIIB distal humerus fracture, resulting in a small sample size. Additionally, the location and function of the city in which the study was conducted, as well as the occupational characteristics of the patients, may have influenced the results.

### 4.2 Surgical Challenges

Surgical treatment is often necessary for low distal humerus fractures due to the absence of muscle or ligament attachment to the fracture fragments and the challenges of closed reduction. Duckerley type IIIB fractures, characterized by the separation of both the capitellum and trochlea with posterior comminution, present significant surgical difficulties. Some cases may exhibit sagittal and axial plane fracture lines, leading to the formation of multiple small fragments within the capitellum and trochlea [10] (Figure 4).

### 4.3 Surgical Tips

Successful management of Duckerley type IIIB fractures requires careful surgical planning and precise execution to restore joint integrity and function. Due to the high number of fracture fragments, fragment excision may simplify the procedure and reduce surgical time; however, it can compromise elbow stability and lead to complications such as postoperative pain, traumatic arthritis, and mobility dysfunction [21, 22]. Thus, preserving and reconstructing the articular surface through open reduction and internal fixation (ORIF) is recommended whenever feasible.

When selecting a surgical approach for these patients, we used the olecranon osteotomy approach to preserve the medial and lateral collateral ligaments as much as possible and to provide greater exposure of the articular surface. This seems to be a consensus [11, 23, 24, 25, 26]. Based on the experience of subchondral placement of Kirschner wires in comminuted fractures of the talus (Hawkins III), we used Kirschner wires to immobilize small fragments in intra‐articular fractures of the talus, providing a good restoration of the articular surfaces and adequate fixation strength [13]. In this study, we successfully used the same technique to immobilize smaller fracture fragments. It is important to avoid repetitive drilling when placing Kirschner wires to prevent fixation failure caused by pin extraction. The placement of Kirschner wires should be carefully considered to ensure easy handling in case of pin withdrawal or bone fragment resorption. In this study, one case of Kirschner wire displacement occurred, which was subsequently removed after the internal fixation was removed. In addition, microplates were utilized to support and cover the small fracture fragments of the posterior humerus, providing enhanced stability to the fracture site through multiangle fixation. It is important to note that careful attention must be paid to the placement of the plate to prevent impingement of the radial head during activity.

### 4.4 Postoperative Management and Rehabilitation

**FIGURE 8:**
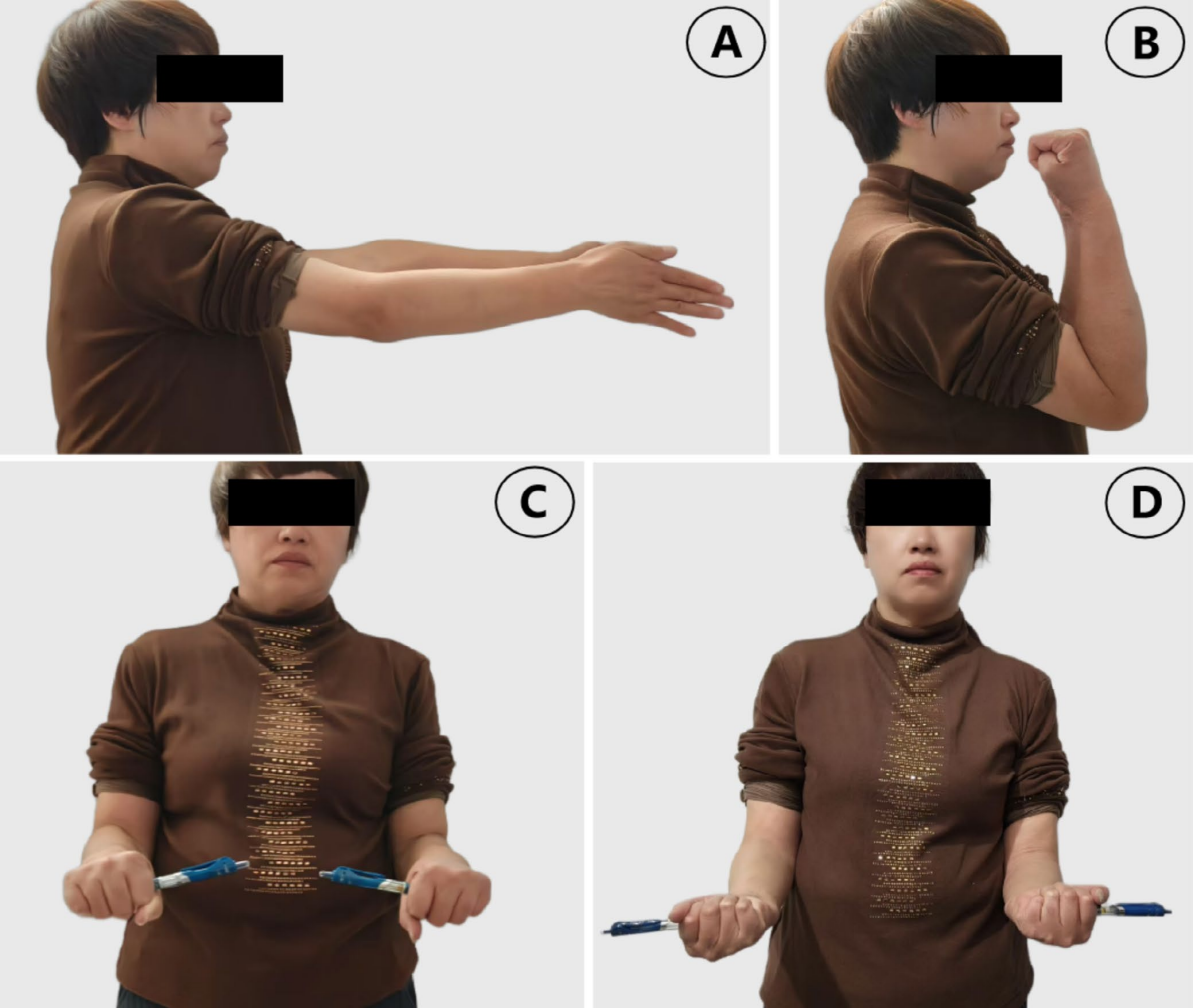
(A–D) Female, 54 years old. Good function was achieved at the 48‐month follow‐up (36 months after internal fixation removal operation). (A) Extension, (B) flexion, (C) pronation, and (D) supination.

For 6 weeks, the patient's elbow was immobilized using a brace that maintained a 90° flexion. During this period, activities were limited to those of unaffected joints to avoid any risk of postoperative internal fixation failure. Following the 6‐week period, the rehabilitation reinforcement exercise program was initiated, incorporating continuous passive motion and physical therapy. Between 8 weeks and 6 months, while maintaining the previous training, resistance exercises were progressively introduced. It is worth noting that this approach differs from the mainstream technology approach, where early range of motion training is typically initiated within 2 weeks [15, 16]. The rationale behind our actions is that, due to the severe comminution of the fracture block, it is recommended to maintain firm functional positional fixation until the fracture site is stabilized than to lose the fixture and have to be saved by total elbow arthroplasty (TEA) [27]. Certain academics likewise concur with the notion that the immobilization time depends on the type of fracture and the stability of the internal fixation [28, 29, 30, 31, 32]. This study found that patients did not have an increased risk of internal fixation failure and ossifying myositis after 6 weeks of postoperative cast braking (Figure 8), and also did not undergo secondary release surgery, which may be attributed to the aforementioned reasons.

It has been noted that TEA is considered a safer and more effective alternative to ORIF in the treatment of some elderly distal humerus fractures with osteoporosis [10, 25]. However, such patients were not included in the present study due to the epidemiological characteristics of the sample. Nevertheless, given the complexity of managing severe low distal humerus fractures with osteoporosis, we believe that TEA may be an acceptable alternative.

Complications of Duckerley type IIIB distal humerus fracture are not uncommon due to their unique nature. These complications include early neurovascular injury, osteofascial compartment syndrome, infection, osteonecrosis, traumatic arthritis, heterotopic ossification, fracture nonunion, delayed healing, loosening of the internal fixation, dislocation of the joint, neuroinflammation, stiffness, and pain syndromes. It is important to note that these complications are objective and supported by evidence. The study found that one case experienced displacement of the Kirschner wires, two cases were left with elbow dysfunction, and the remaining cases had no other complications. The small sample size may have contributed to these results. However, completing the anatomical repositioning of the articular surfaces as much as possible intraoperatively and using Kirschner wires for buried fixation of very small fracture fragments is also important in reducing the incidence of traumatic arthritis.

### 4.5 Strengths and Limitations

This study presents several strengths: a key strength is the introduction of a novel surgical technique for the treatment of Duckerley type IIIB fractures. The use of Kirschner wires to immobilize small fracture fragments, combined with microplates for enhanced stability, represents a creative approach that has shown promise in reducing complications such as traumatic arthritis and fixation failure. Another strength lies in the comprehensive clinical, functional, and radiological assessment of outcomes, which allows for a satisfactory evaluation of the effectiveness of the surgical technique.

However, there are some limitations to consider. This study is retrospective, with insufficient follow‐up duration and a small sample size, which may not be representative of all Duckerley type IIIB distal humerus fractures. The findings may require larger cohort studies with longer follow‐up and prospective studies for further validation.

### 4.6 Prospect of Clinical Application

Although the combination of submerged Kirschner wires with plate or screw fixation has shown promising results, there is room for further optimization. Large multicenter clinical studies are essential. Conducting multicenter trials will help establish standardized protocols, improve surgical outcomes across diverse populations, and ensure broader adoption of the technique in clinical settings. Meanwhile, the optimal duration of postoperative fixation and the time to start rehabilitation training are still worth noting; future research should aim to identify the most effective balance between immobilization and early rehabilitation, tailoring protocols to patient‐specific factors such as bone quality, fracture severity, and fixation stability. Comparative studies between different fixation methods will also provide valuable insights into selecting the most appropriate approach for various fracture patterns.

## 5 Conclusions

The combination of submerged Kirschner wires and plate or submerged screw fixation techniques has satisfactory advantages in terms of fracture reduction, maintenance of the position of internal fixation, and postoperative recovery when treating Duckerley type IIIB distal humerus fracture, which is a rare and complex fracture.

## Author Contributions

**Zhou‐Feng Song:** designed the study, wrote the text, approved the final version for submission. **Wei‐Qiang Zhao:** cases collected, collected and analyzed the data, approved the final version for submission. **Zeng‐Li Zhang:** cases collected. **Jie‐Feng Huang:** designed the study, conceptualization, wrote and modified the text, prepared the figures, approved the final version for submission.

## Ethics Statement

Ethics committee approval was received for this study from the Institutional Review Board of the First Affiliated Hospital of Zhejiang Chinese Medical University (No. 2024‐KLS‐689‐02).

## Consent

Informed consent was obtained from all individual participants included in the study.

## Conflicts of Interest

The authors declare no conflicts of interest.

## Data Availability

The data are available from the authors upon reasonable request.
