# Correction to “Surgical Outcomes of Olecranon Osteotomy Approach Combined With Submerged Kirschner Wires and Plate Fixation for Duckerley IIIB Distal Humeral Coronal Shear Fractures”

**DOI:** 10.1111/os.70177

**Published:** 2025-09-18

Z. F. Song, W. Q. Zhao, Z. L. Zhang, and J. F. Huang, “Surgical Outcomes of Olecranon Osteotomy Approach Combined With Submerged Kirschner Wires and Plate Fixation for Duckerley IIIB Distal Humeral Coronal Shear Fractures,” *Orthopaedic Surgery* 17, no. 4 (2025): 1255–1264. https://doi.org/10.1111/os.70005.

In the published article, the word **“Duckerley”** (or occasionally **“Dubbrey's”**) was mistakenly used in place of the correct term **“Dubberley”** in several instances as specified below:

In the Title, the text “**Duckerley IIIB Distal Humeral Coronal Shear Fractures**” was incorrect. This should have read: “**Dubberley IIIB Distal Humeral Coronal Shear Fractures**”.

In the Abstract, Objective and Methods section, the text “**Duckerley type IIIB distal humerus fractures**” was incorrect. This should have read: “**Dubberley type IIIB distal humerus fractures**.”

In paragraph 2 of the “Introduction” section, the text “**Duckerley et al.**” was incorrect. This should have read: “**Dubberley et al.**” The phrase “**Duckerley type IIIB fractures**” was incorrect. This should have read: “**Dubberley type IIIB fractures**.”

In paragraph 4 of the “Introduction” section, the text “**Duckerley type IIIB distal humerus fractures**” was incorrect. This should have read: “**Dubberley type IIIB distal humerus fractures**.”

In Figure 1 caption, text “**Dubbreys classification**.” was incorrect. This should have read: “**Dubberley classification**.”

In paragraph 2 of the “Materials and Methods” section, the text “**Duckerley type IIIB distal humerus fractures**” was incorrect. This should have read: “**Dubberley type IIIB distal humerus fractures**.”

In paragraph 4 of the “Materials and Methods” section, the text “**Duckerley type IIIB**” was incorrect. This should have read: “**Dubberley type IIIB**.”

In Figure 2 caption, the text “**Duckerley type IIIB distal humerus fractures**” was incorrect. This should have read: “**Dubberley type IIIB distal humerus fractures**.”

In paragraph 1 of the “Discussion” section, the text “**Duckerley type IIIB distal humerus fractures**” was incorrect. This should have read: “**Dubberley type IIIB distal humerus fractures**.”

In paragraph 2 of the “Discussion” section, the text “**Duckerley type IIIB distal humerus fractures**” was incorrect. This should have read: “**Dubberley type IIIB distal humerus fractures**.”

In paragraph 3 of the “Discussion 4.2 Surgical Challenges” section, the text “**Duckerley type IIIB fractures**” was incorrect. This should have read: “**Dubberley type IIIB fractures**.”

In paragraph 4 of the “Discussion 4.3 Surgical Tips” section, the text “**Duckerley type IIIB fractures**” was incorrect. This should have read: “**Dubberley type IIIB fractures**.”

In paragraph 8 of the “Discussion” section, the text “**Duckerley type IIIB distal humerus fractures**” was incorrect. This should have read: “**Dubberley type IIIB distal humerus fractures**.”

In paragraph 9 of the “Discussion 4.5 Strengths and Limitations” section, the text “**Duckerley type IIIB fractures**” was incorrect. This should have read: “**Dubberley type IIIB fractures**.”

In paragraph 10 of the “Discussion” section, the text “**Duckerley type IIIB distal humerus fractures**” was incorrect. This should have read: “**Dubberley type IIIB distal humerus fractures**.”

In the “Conclusions” section, the text “**Duckerley type IIIB distal humerus fractures**” was incorrect. This should have read: “**Dubberley type IIIB distal humerus fractures**.”

We apologize for this error.

